# Methylprednisolone alleviates cognitive functions through the regulation of neuroinflammation in Alzheimer’s disease

**DOI:** 10.3389/fimmu.2023.1192940

**Published:** 2023-05-01

**Authors:** Yuan Sun, Jinran Li, Nan A, Zhaoxing Li, Wenyi Zhong, Long Chen, Sai Liu, Bocheng Zhang, Zheying Zhu, Xinuo Li

**Affiliations:** ^1^ Jiangsu Provincial Key Laboratory of Drug Metabolism and Pharmacokinetics, State Key Laboratory of Natural Medicines, China Pharmaceutical University, Nanjing, China; ^2^ Digestive Endoscopy Department, The First Affiliated Hospital with Nanjing Medical University and Jiangsu Province Hospital, Nanjing, China; ^3^ State Key Laboratory of Natural Medicines and Jiangsu Key Laboratory of Drug Design and Optimization, China Pharmaceutical University, Nanjing, China; ^4^ Department of Organic Chemistry, China Pharmaceutical University, Nanjing, China; ^5^ School of Pharmacy, The University of Nottingham, Nottingham, United Kingdom

**Keywords:** Alzheimer’s disease, neuroinflammation, methylprednisolone, cognitive function, microglial activation

## Abstract

Alzheimer’s disease (AD) is a progressive neurodegenerative disease and linked to abnormal deposition of amyloid-β (Aβ), neurofibrillary tangles (NFTs), synaptic dysfunction, and neuroinflammation. Despite significant progress in unravelling the pathogenesis of AD, currently main therapeutic interventions is limited to symptomatic alleviation. Methylprednisolone (MP), a synthetic glucocorticoid, is recognized for its extensive anti-inflammatory properties. Our study assessed the neuroprotective effect of MP (25 mg/kg) administration to an Aβ_1-42_-induced AD mouse model. Our findings demonstrate that MP treatment can ameliorate cognitive impairment in Aβ_1-42_-induced AD mice and suppress microglial activation in the cortex and hippocampus. RNA-Sequencing analysis reveals that MP ultimately rescues cognitive dysfunction through improving the synapse function and inhibiting the immune and inflammatory processes. Our study suggests that MP could be a promising drug alternative for the treatment of AD, either alone or in combination with other existing drugs.

## Introduction

Alzheimer’s disease (AD) is a neurodegenerative brain condition leading to a progressive decline of memory and other brain functions, which include abnormal deposition of amyloid-β (Aβ), neurofibrillary tangles (NFTs), neuronal loss, synaptic dysfunction, neurotransmitter imbalance and neuroinflammation (1). Such outcome forms a chronic disease that affects roughly 8% of the world’s population — an astonishing 50 million people (2, 3). AD has become one of the leading causes of death worldwide. So far, there are no disease-modifying treatments for AD (4, 5).

Neuroinflammation is a prominent feature in the pathogenesis of AD (6), representing a defense mechanism that initially protects the brain by inhibiting various pathogens (7, 8). However, persistent inflammatory responses involving microglia can lead to neurodegenerative diseases (9). Microglial cells, the resident macrophages of the central nervous system, can recognize damaged cells and foreign stimuli through their surface receptors, exerting immune effects by producing cytotoxic factors and proinflammatory cytokines (10). It was reported that microglia activation and the subsequent release of proinflammatory factors play a significant role in neuronal damage (11). There is compelling evidence in AD that Aβ deposition is associated with a local inflammatory response initiated by microglia activation (12). Increased levels of proinflammatory cytokines are associated with an increasing rate of cognitive decline in patients with AD (13). Taken together, alleviating neuroinflammation by inhibiting the overactivation of microglia is a potential treatment for AD.

Synaptic loss is an important pathologic feature of AD, and the strong relationship between synapse loss and microglia abnormality has been well documented. Previous findings had documented that the complement-dependent pathway and microglia that prune excess synapses in development are inappropriately activated and mediate synapse loss in AD (14). The microglial innate immune receptor TREM2, the deficiency of which aggravates Aβ accumulation and neuron damage in AD (15), is essential for microglia-mediated synaptic refinement during the early stages of brain development (16), indicating abnormal microglia plays a key role in mediating synapse loss in AD (17).

Glucocorticoids (GCs) have been used clinically as potent anti-inflammatory agents for decades (18). Prednisolone and methylprednisolone (MP), synthetic GCs, are frequently used to manage autoimmune, inflammatory, and allergic disorders, including rheumatoid arthritis (RA), inflammatory bowel disease, lupus erythematosus, transplant rejection, and asthma (19).

Methylprednisolone (MP) has the anti-inflammatory activity more than three times than that of prednisolone (20) and is more effective than dexamethasone in reducing complications associated with hospitalized hypoxic COVID-19 patients and following major hepatectomy (21, 22). Moreover, studies have indicated that MP can decrease inflammation and aid in the recovery of neurological function in various conditions, including spinal cord injury (23, 24), crush-type sciatic nerve injury (25), and lung injury induced by brain death (26, 27). MP exerts multiple anti-inflammatory effects, such as inhibiting the activation of T cells, dampening the inflammatory cytokine cascade, decreasing immune cells’ extravasation into the central nervous system, and promoting apoptosis of activated immune cells (28). Increasing evidence suggests that MP provides neuroprotection by activating the Wnt/β-catenin (29) and PI3K-Akt signaling pathways (30) while inhibiting glial activation (31).

Considering the established anti-inflammatory properties of MP and the significant role of neuroinflammation in cognitive dysfunction and neuronal damage, we postulate that MP may have potential therapeutic benefits in AD due to its anti-inflammatory action. Nonetheless, it remains unclear whether MP can alleviate learning and memory deficits and reduce neuroinflammation in AD.

The present study aimed to investigate the potential role of MP in Aβ_1-42_-induced mice of AD. Our findings indicated that MP treatment could improve the cognitive impairment induced by Aβ_1-42_ in mice by suppressing the excessive microglia activation.

## Results

### MP alleviates cognitive dysfunctions in Aβ_1-42_-induced mice

**Figure 1:**
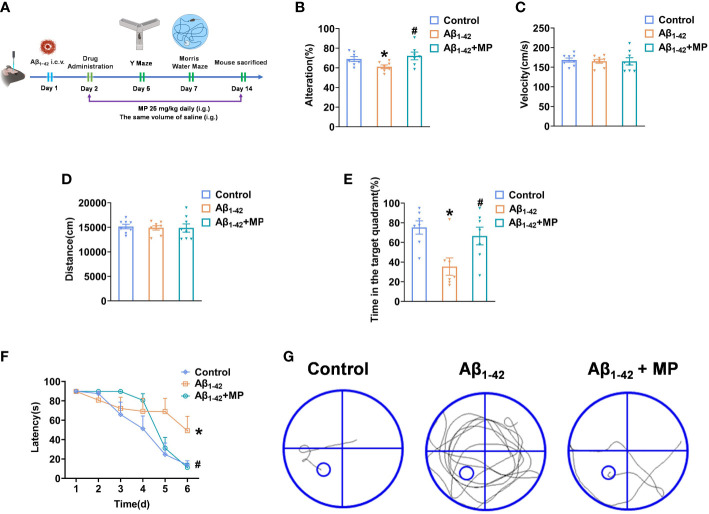
MP can alleviate cognitive dysfunction in Aβ_1-42_-induced mice. Once injected with Aβ_1-42_ (10 ug, 5 uL, i.c.v.), the mice were treated with MP (25 mg/kg, i.g.). **(A)** Schematic of experimental design. **(B)** The spontaneous alternation performance in the Y-maze test. **(C)** Path length of each group. **(D)** Average swimming speed of each group. **(E)** Spent time in the target quadrant at the last exploration trial. **(F)** The latency to find the target platform during training trials and the last exploration trail. **(G)** The swimming paths of mice finding the hidden platform at the last exploration trial. Data are shown as mean ± SEM (n=10). *P < 0.05 vs. control group; ^#^P < 0.05 vs. Aβ_1-42_-induced group.

To investigate whether MP could alleviate cognitive dysfunction in AD, behavioral experiments were performed to evaluate the potential effect of MP on learning and memory abilities in mice treated with Aβ_1-42_ using the Y maze and Morris Water Maze **Figure 1A**. Y-maze test was conducted on all mice to assess the short-term spatial working memory, and the result demonstrated that MP treatment could attenuate the Aβ_1-42_-induced reduction in spontaneous alternation (**Figure 1B**), indicating an improvement in spatial recognition. Our results showed that Aβ_1-42_ could significantly impair spatial recognition in mice, and MP could improve the recognition deficiency.

The Morris Water Maze was subsequently used to explore the learning and memory capacity of mice. We recorded the distances and average swimming speeds to eliminate the possibility that spatial learning deficits in mice were due to movement disorders (**Figures 1C, D**). No significant difference among all groups indicated that all mice had normal motor ability. The MP-treated Aβ_1-42_-induced mice spent more time in the target quadrant than the Aβ_1-42_-induced group and took less time to locate the target platform during the last exploration trial (**Figures 1E, F**). During the probe trial, mice treated with MP simplified the swimming tracks to find the target platform compared to the Aβ_1-42_-induced group (**Figure 1G**). Above all, these results showed that MP could effectively alleviate cognitive dysfunction in Aβ_1-42_-induced mice.

### MP inhibits the excessive activation of microglia in the cortex and hippocampus of Aβ_1-42_-induced mice

**Figure 2:**
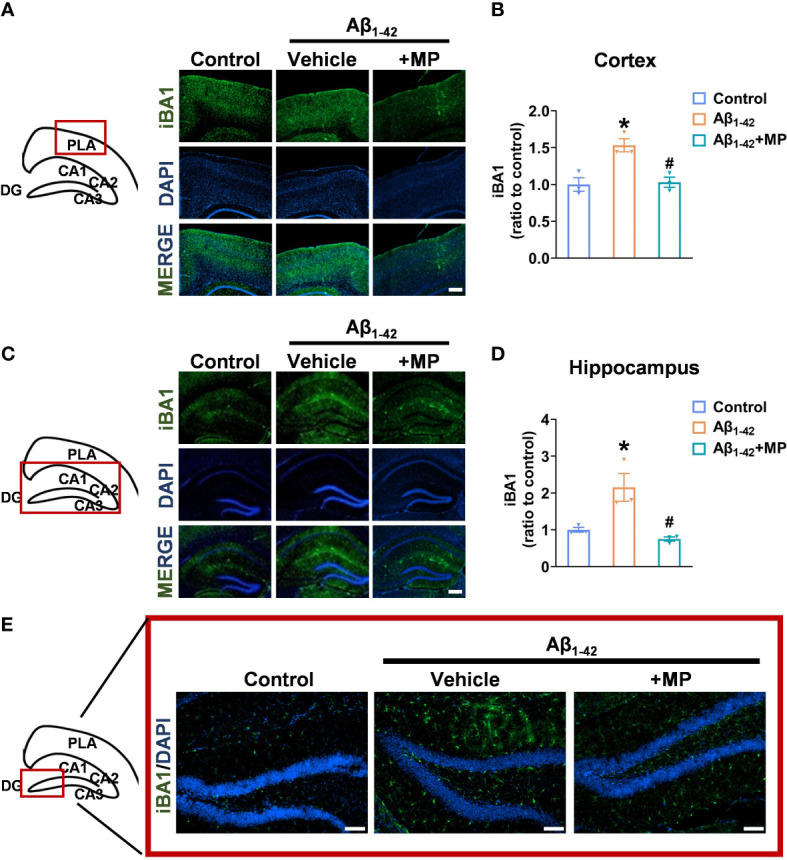
MP inhibits the excessive activation of microglia in the cortex and hippocampus of AD mice. **(A)** Representative fluorescence micrographs showing iBA1 expression in the cortex (Scale bar, 1000 μm). **(B)** Quantification of the total number of iBA1^+^ cells in the cortex (n = 3). **(C)** Representative fluorescence micrographs showing iBA1 expression in the hippocampus (Scale bar, 1000 μm). **(D)** Quantification of the total number of iBA1^+^ cells in the hippocampus. (n = 3). **(E)** Representative fluorescence micrographs showing iBA1 expression in DG (Scale bar, 200 μm). Data are shown as mean ± SEM (n = 3). *P < 0.05 vs. control group; ^#^P < 0.05 vs. Aβ_1-42_-induced group.

Microglial activation is closely linked to neuroinflammation, and in a hyperactivated state, microglia can generate and discharge inflammatory mediators, leading to neurotoxicity. We assessed the degree of microglial activation in the cortex and hippocampus to investigate whether MP would exert its anti-inflammatory effect in Aβ_1-42_-induced mice. Immunofluorescence analysis showed that Aβ_1-42_ induced excessive microglial activation in the cortex (**Figures 2A, B**) and hippocampus (**Figures 2C–E**), whereas MP treatment markedly reduced the number of activated microglia. The results indicated that MP played an anti-inflammatory role by inhibiting the microglia activation in Aβ_1-42_-induced mice.

### MP regulates the expression of mRNA related to synaptic function, immunity, and inflammation

**Figure 3:**
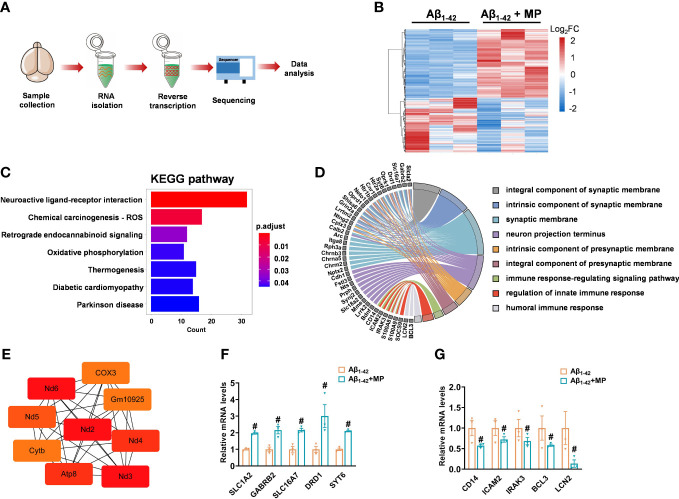
MP altered mRNA expression profile of Aβ_1-42_-induced mice. **(A)** Schematics of the experimental design. **(B)** Heatmap of DGEs in MP-treated Aβ_1-42_-induced mice and the control Aβ_1-42_-induced mice. **(C)** KEGG annotation for the differentially expressed genes. **(D)** GO enrichment analysis of DEGs and functional analysis for genes. **(E)** Hub genes identified from the PPI network based on the MCC algorithm in cytoHubba. **(F)** Quantified image shows the mRNA expression level of genes associated with synaptic function. **(G)** Quantified image shows the mRNA expression level of genes associated with immune response. Data are shown as mean ± SEM (n = 3). ^#^P < 0.05 vs. Aβ_1-42_-induced group.

The brains samples prepared from MP-treated Aβ_1-42_-induced mice and the control Aβ_1-42_-induced mice were carried out by RNA sequencing to determine whether MP could alter mRNA expression (**Figure 3A**). A total of 556 differentially expressed genes (DEGs) was identified between the two groups, of which 309 were upregulated and 247 were downregulated with a threshold of adjusted P-value <0.05 (**Figure 3B** and **Supplementary Table 1**). In order to speculate the possible function affected by MP, KEGG annotation and GO analysis was carried out on these DEGs, and some significant terms were enriched on neuroactive ligand-receptor interaction, synaptic function, neuron projection terminus and immune response (**Figures 3C, D**). Among them, SLC1A2, GABRB2, SLC16A7, DRD1, SYT6, CD14, ICAM2, IRAK3, BCL3 and LCN2 were interestingly found in GO results. Subsequently, protein-protein interaction was performed, and hub genes were identified by cytoHubba based on the MCC algorithm, including ND3, ND2, ATP8 and COX3, which are associated with mitochondrial function (**Figure 3E**). The results showed that some genes associated with synaptic function were upregulated while several other genes involved in immune response were downregulated (**Figures 3F, G**). These results indicated that MP might alleviate cognitive dysfunction by improving the synapse function and inhibiting the immune and inflammatory processes.

**Figure 4:**
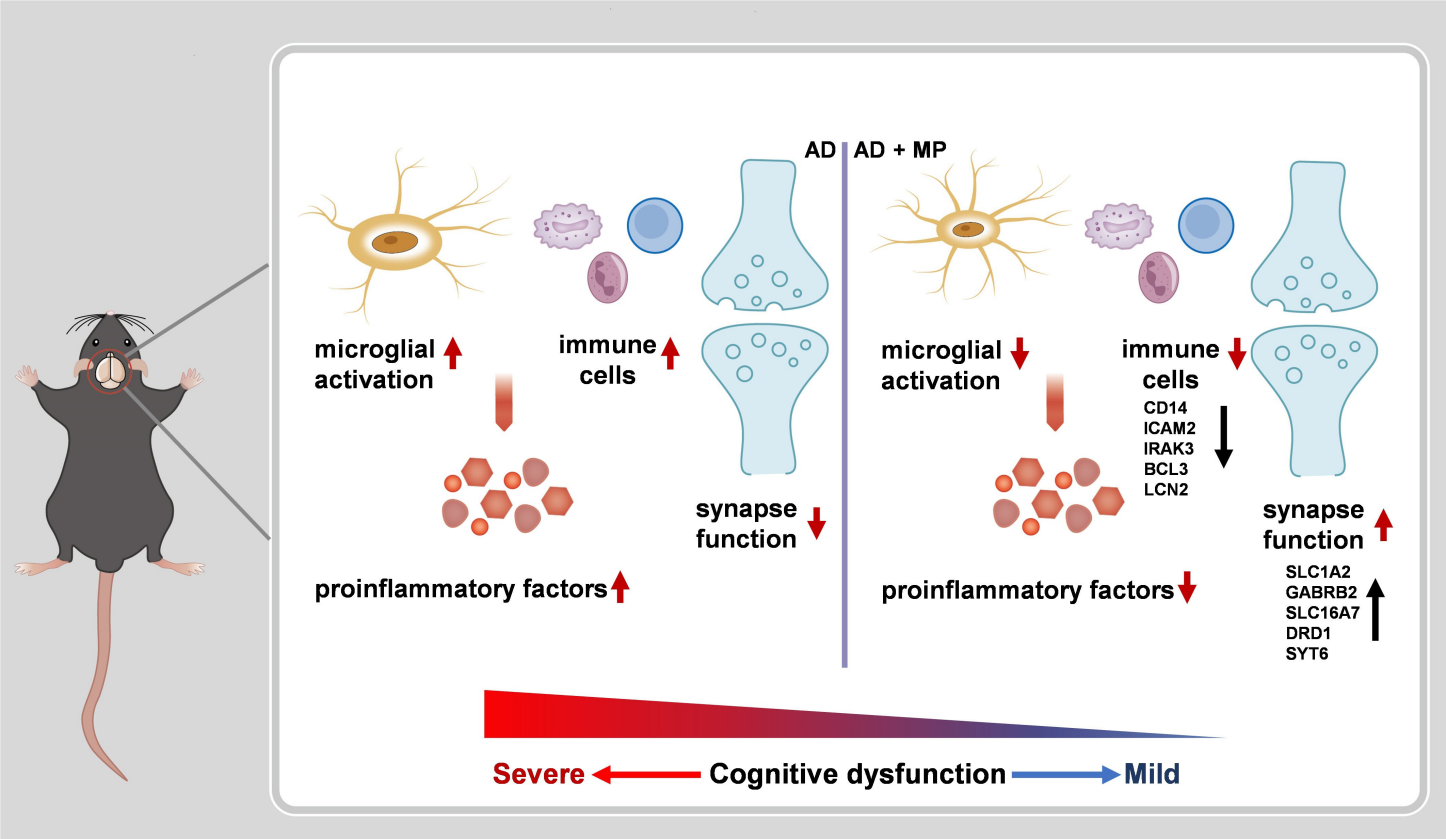
A schematic representation of MP treatment alleviating cognitive dysfunction in AD nice. The administration of MP suppresses microglial activation and immune response, causing proinflammatory factors inhibition. Meanwhile, MP improves synaptic function by regulating the expression of the related genes. Altogether MP ameliorates recognition deficiency induced by Aβ_1-42_ in mice.

Our results demonstrated that treatment MP treatment could significantly alleviate cognitive dysfunction in Aβ_1-42_-induced mice through significant inhibition of microglial activation. Furthermore, RNA-seq revealed that MP could improve the synapse function and inhibit the immune and inflammatory processes. These findings are summarized in **Figure 4**.

## Discussion

In this study, we investigated whether MP could be a therapeutic option for Alzheimer’s disease (AD) by leveraging its anti-inflammatory properties in Aβ_1-42_-induced mice. The results showed that MP could improve the behavioral performance of Aβ_1-42_-induced mice in the Y maze and Morris Water Maze, indicating that MP has potential therapeutic application for alleviating cognitive impairment in mice suffering from AD. Furthermore, our analysis of mouse brain tissues through immunofluorescence staining revealed that MP treatment could inhibit cortical and hippocampal microglia activation, significantly attenuating neuroinflammatory responses. RNA sequencing results further indicated that MP might alleviate cognitive dysfunction by improving the function of synapses and neurons. These results suggest that MP may be a potential drug candidate for the treatment of AD.

Alzheimer’s disease is widely recognized for its hallmark features of progressive accumulation of senile plaques consisting of Aβ, neurofibrillary tangles, synapse dysfunction, and neuroinflammation in the brain. Neuroinflammation refers to the inflammatory responses occurring in the brain, and it is implicated as a significant contributor to the development and progression of AD (32). Microglia, a type of glial cell, is closely related to the occurrence of neuroinflammation. When an acute inflammatory injury occurs in the brain, the microglia have an initial defensive response to repair the tissue damage. However, when the stimulus persists for an extended period, it can lead to chronic inflammatory conditions, negatively impacting the central nervous system (CNS) and exacerbating neuronal dysfunction (33). Activated microglia change to an amoeboid shape, migrate to sites of inflammation, and secrete proteins such as cytokines, chemokines, and reactive oxygen species. These molecules released by microglia can lead to synaptic plasticity and learning and memory deficits associated with AD (34, 35). Therefore, inhibition of microglia activation and eliminating neuroinflammation may be practical strategies to attenuate AD progression while simultaneously relieving symptoms associated with the disease.

MP is a widely applied glucocorticoid treatment for various clinical conditions, including arthritis, allergies, multiple sclerosis, and acute spinal cord injury. With its high oral bioavailability and capacity to cross the blood-brain barrier, the efficacy of MP has been confirmed *via* the facilitation of neurological functioning recovery in various disorders. However, whether MP can be a possible therapeutic alternative for AD remains unclear.

Our findings refined the current understanding of the positive role of MP in neuroinflammation that may provide a potential therapeutic for AD. The immunofluorescence results showed that MP could inhibit the excessive activation of microglia in the cortex and hippocampus, exerting anti-inflammation function. We performed the GO analysis based on the differentially expressed mRNA profile to explore the enriched signaling pathway changed by MP. GO analysis revealed that MP suppressed the immune process, and some genes associated with the immune process were significantly downregulated, including CD14, ICAM2, IRAK3, BCL3 and LCN2, which consisted of inhibiting activated microglia, further confirming the anti-inflammation effect of MP. Besides, MP could promote the synaptic membrane function, and the genes associated with synaptic membrane were significantly upregulated, including SLC1A2, GABRB2, SLC16A7, DRD1 and SYT6, indicating that MP may have a protective effect on the synapse. Consistent with our finding, a study showed that MP could improve neuromuscular transmission deficits *via* contributing to synaptic vesicle redistribution (36), confirming that MP may improve synapse function.

Nonetheless, some limitations in our research must be acknowledged. The exact mechanism by which MP exerts its neuroprotective effect remains unclear, and thus further research would be necessary to uncover the exact mode of step to update our current understanding of the drug’s effectiveness in treating AD, by further verification certified to uncover the molecular mechanism underlying the effects of MP in AD. Our RNA-Seq analysis results indicate that the protective effect of MP may be associated with improving the function of synapses and neural interaction, which remains to be explored further to find the specific molecular mechanism. Given the possibility of adverse side effects associated with hormonal drugs, careful consideration of dosage is necessary when using MP in treating AD. Notably, higher doses of MP were not tested in our study, and further assessments of potential toxicity, including acute and long-term toxicity testing, should be undertaken to determine the safety limitations of the drug in treating AD.

It is known that the drugs currently available for the treatment of AD cannot halt the disease’s pathological progression during its middle and late stages. Using MP as an adjuvant drug combined with existing in clinical may significantly enhance the efficacy of such drugs in treating AD. It is worth noting that the AD drugs in clinical are broadly classified into two categories, including cholinesterase inhibitors and a NMDA receptor antagonist, each class of which has a different mechanism of action from that of MP, indicating that a combination therapy strategy involving MP holds enormous potential in significantly improving treatment outcomes for AD.

In conclusion, our study on an Aβ_1-42_-induced mouse model conclusively demonstrates that MP benefits AD by effectively suppressing neuroinflammation and limiting microglial activation to promote cognitive improvement. These results suggest that using MP in treating AD may be a promising therapeutic strategy, either by a single candidate or in combination with other existing drugs.

## Materials and methods

### Animals and manipulations

All male C57/BL6 mice (6 weeks old, weight 18-22 g) were purchased from Changzhou Cavens Laboratory Animal Company. Animal culture and procedures were according to the regulations for the management of experimental animals issued by the Ministry of Science and Technology of the People’s Republic of China and approved by the Pharmaceutical Laboratory Animal Center of China Pharmaceutical University.

### Aβ_1-42_ injection and drug intervention

Recombinant human Aβ_1−42_ peptide was obtained from Beyotime Biotechnology (China). Aβ_1−42_ peptide was dissolved in sterile PBS at a concentration of 2 mg/mL and incubated for 5 days at 37°C to induce Aβ_1−42_ aggregation. All mice were divided into three groups as follows (n = 10 per group): vehicle as the blank control group, Aβ_1−42_ (intracerebroventricular injection, i.c.v.) as the model group, Aβ_1−42_ (intracerebroventricular injection, i.c.v.) + MP (25 mg/kg, intragastric administration, i.g.) group. Mice were injected into the lateral ventricle with 5 uL oligomerized Aβ_1−42_ peptide on the first day of the experiment through the brain stereo-positioning instrument. The control group was injected with the same volume of saline in the same way. MP (dissolved in saline) was intragastrically administrated during 2-14 days. Control and model group mice were gavaged with only normal saline.

### Y maze

The Y maze was mainly used to assess spatial working memory in rodents. The maze consisted of a symmetrical Y-shaped device with three identical arms spaced 120^°^ apart and made of black plexiglass. The mice were dropped from one arm and allowed to explore the whole maze for 10 min. The order and total number of times the animal entered each arm were recorded. Normal mice exhibited high levels of spontaneous alternating behavior with successive visits to different open arms (e.g., A–B–C–A–B). The number of correct alternating responses was counted, and the spontaneous alternating rate was calculated.

### Morris water maze (MWM)

The MWM was often used to evaluate the spatial learning and memory abilities of mice. The whole maze consists of a pool and an underwater platform. The pool (60 cm radius, 45 cm height) was divided into four quadrants randomly and the platform (1 cm under the water level) was on the center of one quadrant. The pool was filled with water, which is made opaque by the addition of titanium dioxide. The water temperature was maintained at about 25 °C.

Behavior experiments were conducted 7 days after drug administration, including training for 5 days and testing the spatial learning and memory ability at day 6. For the training trials daily, mice were placed opposite the quadrant where the platform was located, facing the pool wall, and allowed to swim for finding the platform which was 1 cm above the water. If the mouse reached the platform within 90 s, it was allowed to rest there for 10 s. Otherwise it was manually guided to the platform and rested on the platform for 10 s to remember the position. At day 6 of the experiment, the platform was hidden 1 cm under the water and the space exploration trial was performed. The latency, path length, swimming velocity, target quadrant residence time and traveled trajectory were recorded by the computer daily.

### Brain tissue preparation

Mice were sacrificed under deep anesthesia at day 14 and were handled as follows: For immunofluorescence analysis, mice were perfused with frozen PBS (PH 7.4) transcardially and followed by perfusing with 4% paraformaldehyde (PFA) dissolved in PBS for tissue fixation. After cardiac perfusion, the brains were harvested, fixed in 4% PFA and protected from light at 4°C until use. For RNA sequencing, mice were transcardially perfused with frozen PBS only and the brains were stored at -20°C.

### Mice brain slices and immunofluorescence

The brain tissue fixed for at least 24 hours was transferred into 30% sucrose solution for dehydration for 48 hours. After sinking to the bottom, the brains were cut into 30 μm-thick coronal slices using a freezing microtome (Leica, CM1950), and the brain slices with an intact hippocampus were stored in the freezing solution (PBS: ethylene glycol: glycerin = 5:3:2) and stored at -20 °C.

The brain slices were washed 3 times in PBS for 5 min each time, following by treating in 0.1% Triton X-100 (Beyotime Biotechnology, ST795) for 20 min. Then the slices were placed in 10% donkey serum (Solarbio, SL050) diluted with PBS and blocked for 1 h at room temperature. After incubated with the primary antibody [rabbit anti-iBA1 (Fujifilm, 019-19741, 1:600)] overnight at 4°C, the slices were washed 3 times in PBS. Then the secondary antibody [Goat Anti-Rabbit IgG H&L (Alexa Fluor^®^ 488) (Abcam, ab150077, 1:500)] was applied for 1 h at room temperature, and the slices were washed 3 times in PBS. The nuclei were finally counterstained for 10 min with 1 μg/ml DAPI (Beyotime Biotechnology, C1002), and slides were mounted with anti-fluorescence quenching sealing tablets. The slides were visualized with a fluorescence microscope (BioTek, Cytation5), and images were collected with a MicroImaging System (BioTek, Cytation5). Finally, the images acquired were analyzed using Image J software.

### RNA isolation and RNA sequencing

Total RNA was isolated from mice brain tissues by using RNA isolater Total RNA Extraction Reagent (Vazyme, R401-01) and was submitted to Frasergen Genomic Medicine (Wuhan, China) for RNA-sequencing (RNA-seq). Equal amount of RNAs from 3 animal group were pooled and used for RNA-seq analysis. The raw RNA-seq reads were mapped to the mouse genome (mm10) by HISAT2 (2.2.1) and then were quantified with FeatureCounts and the resulting quantization file was further evaluated with R (v4.2.2). Subsequently, DESeq2 package was used to identify differentially expressed genes (DEGs) and we considered genes with P adj < 0.05 and absolute value of log_2_FoldChange > 0.58 as DEGs. ClusterProfiler R package was used to analyze Kyoto Encyclopedia of Genes and Genomes (KEGG) pathway enrichment and Gene ontology (GO) enrichment analysis of DEGs. The screening conditions were adjusted for P < 0.05 and q < 0.05. The protein-protein interaction (PPI) network was built by STRING database (https://string-db.org/), and CytoHubba plugin in Cytoscape (v3.9.0) was used to determine the hub genes.

### Statistical analysis

At least three biological replicates were performed for all experiments to ensure consistency. Data were expressed as means ± SEM, and statistical analyses was performed with GraphPad Prism 8 using Student’s *t*-test. Statistical significance was set at a P value of less than 0.05.

## Data availability statement

The original contributions presented in the study are included in the article/**Supplementary Material**. RNA-seq data that support the findings of this study have been successfully deposited in NCBI Trace Archive NCBI Sequence with the accession number: PRJNA950233 (SRR24013024-SRR24013029), with the link: https://dataview.ncbi.nlm.nih.gov/objects?linked_to_id=PRJNA950233&archive=sr. Further inquiries can be directed to the corresponding authors.

## Ethics statement

The animal study was reviewed and approved by the Pharmaceutical Laboratory Animal Center of China Pharmaceutical University.

## Author contributions

XL and ZZ designed the study. YS conducted the research. JL and NA helped with molecular biology experiments. WZ, LC, and BZ helped with behavioral experiments. ZL, SL, and YS analyzed data and drew relating figures. XL, ZZ, and YS wrote and revised the manuscript. All authors contributed to the article and approved the submitted version.
